# Spatial-temporal patterns and influencing factors for hemorrhagic fever with renal syndrome: A 16-year national surveillance analysis in China

**DOI:** 10.1016/j.onehlt.2024.100725

**Published:** 2024-04-06

**Authors:** Bo Wen, Zurong Yang, Shaolong Ren, Ting Fu, Rui Li, Mengwei Lu, Xiaoang Qin, Ang Li, Zhifu Kou, Zhongjun Shao, Kun Liu

**Affiliations:** a Department of Epidemiology, School of Public Health, Air Force Medical University, Xi'an, People's Republic of China; b Ministry of Education Key Lab of Hazard Assessment and Control in Special Operational Environment, Xi'an, People's Republic of China; c Lintong Rehabilitation and Convalescent Centre, Xi'an, People's Republic of China; d Department of Epidemiology, School of Public Health, Fudan University, Shanghai, People's Republic of China

**Keywords:** Hemorrhagic fever with renal syndrome, Spatial-temporal patterns, Risk factors, China

## Abstract

**Background:**

China is confronted with the significant menace posed by hemorrhagic fever with renal syndrome (HFRS). Nevertheless, the long-term spatial-temporal variations, regional prevalence patterns, and fundamental determinants' mechanisms for HFRS remain inadequately elucidated.

**Methods:**

Newly diagnosed cases of HFRS from January 2004 to December 2019 were acquired from the China Public Health Science Data repository. We used Age-period-cohort and Bayesian Spacetime Hierarchy models to identify high-risk populations and regions in mainland China. Additionally, the Geographical Detector model was employed to quantify the determinant powers of significant driver factors to the disease.

**Results:**

A total of 199,799 cases of HFRS were reported in mainland China during 2004–2019. The incidence of HFRS declined from 1.93 per 100,000 in 2004 to 0.69 per 100,000 in 2019. The incidence demonstrated an inverted U-shaped trend with advancing age, peaking in the 50–54 age group, with higher incidences observed among individuals aged 20–74 years. Hyperendemic areas were mainly concentrated in the northeastern regions of China, while some western provinces exhibited a potential upward trend. Geographical detector model identified that the spatial variations of HFRS were significantly associated with the relative humidity (Q = 0.36), forest cover (Q = 0.26), rainfall (Q = 0.18), temperature (Q = 0.16), and the surface water resources (Q = 0.14).

**Conclusions:**

This study offered comprehensive examinations of epidemic patterns, identified high-risk areas quantitatively, and analyzed factors influencing HFRS transmission in China. The findings may contribute to the necessary implementations for the effective prevention and control of HFRS.

## Highlights

• The HFRS epidemic in China showed a declining trend and appeared to be approaching stability.
• The northern provinces of Heilongjiang and Shaanxi have always been high incidence areas.
• The incidence of HFRS showed an inverted U-shape with age, peaking at 50–54 years old.
• Spatial-temporal HFRS variations correlate with relative humidity, rainfall, and forest cover.

## 1 Introduction

Hemorrhagic fever with renal syndrome (HFRS) is a zoonotic disease caused by varying serotypes of Hantavirus (HV), such as Hantaan virus (HTNV), Seoul virus (SEOV), Puumala virus (PUUV), and Dobrava virus (DOBV) [1]. Humans were infected with HFRS primarily through contact with excrement and secretions from infected animals [2]. The global annual incidence of HFRS is estimated to be approximately 60,000–150,000 cases [3], spreading across numerous countries globally. China accounts for 70–90% of the reported cases worldwide [4], with an annual incidence ranging from 20,000 to 50,000 cases, positioning China as the nation bearing the most substantial burden of HFRS globally [5]. China has classified HFRS as a Class B infectious disease for management since 1956 in efforts to strengthen epidemic control. In 2004, a nationwide direct reporting system was established for surveillance, facilitating real-time monitoring of HFRS outbreaks across the country through individual case reports. Furthermore, China has initiated a vaccination program targeting the 16 to 60-year-old population in key regions since 1994. In 2008, a nationwide immunization campaign for HFRS was implemented in prioritized areas to enhance population immunity coverage. Presently, China has successfully implemented preventive and control measures for HFRS, leading to a declining trend in national case numbers [6]. However, cases have been reported in nearly all provinces across the country, showcasing significant regional variations and clusters in high-incidence areas [7], with the epidemic intensifying notably in key high-incidence regions [8]. For instance, in the Wei River Basin spanning Shaanxi and Gansu provinces, reported cases over the past five years accounted for approximately one-fourth of the national total, with the highest incidence nationwide [9].

Additionally, the outbreak and incidence of zoonotic diseases often exhibit significant variations in response to changes in climate, ecology, and socio-economic factors [10]. HFRS, as a climate-sensitive infectious disease carried by rodent hosts and affected by diverse societal influences, is subject to diverse influences encompassing climate conditions, ecological environments, and socio-economic aspects [11]. Several prior studies have investigated the mechanisms underlying the response of influencing factors related to HFRS. For example, regions with high HFRS incidence are frequently located in temperate regions [12], where temperature and precipitation are crucial meteorological factors influencing HFRS outbreaks. Urban economic growth and changes in land cover due to orchard cultivation often lead to alterations in rodent community structures, thereby increasing the risk of HFRS spillover and diffusion from endemic areas [13]. Nevertheless, inadequate research on high-risk HFRS regions in China, spatial-temporal distribution variances, and potential driving factors persist, while the influence of socio-economic, meteorological, and ecological factors in spatial-temporal dynamics lacks thorough verification [14]. Meanwhile, it is paramount to discern spatial-temporal trends of HFRS, evaluate potential high-risk regions, and conduct in-depth analyses of the influence of socio-economic, meteorological, and ecological factors in spatial-temporal distribution disparities.

This study aims to: (1) identify the age groups at potential risk of HFRS and investigate the spatial-temporal heterogeneity of HFRS from 2004 to 2019 in mainland China. (2) analyze the distribution patterns of hot and cold spots and the changing prevalence trends among various provinces. (3) evaluate the underlying factors associated with the prevalence of HFRS in mainland China. These findings will serve as basis for guiding the development of prevention and control strategies for HFRS in different types of epidemic areas.

## 2 Materials and methods

### 2.1 Data collection and management

Data on newly diagnosed cases of HFRS in 31 provincial administrative units of mainland China from January 2004 to December 2019 was obtained from the Public Health Science Data repository of China (http://www.phsciencedata.cn/Share/index.jsp). The repository provides the monthly incidence cases for different age groups from 2004 to 2019, covering 31 provincial-level administrative divisions in China. All cases of HFRS reported in the system were diagnosed by healthcare professionals following the guidelines suggested by the Chinese Health Commission and recorded in the Notifiable Infectious Diseases Reporting Information System (NIDRIS) (https://weekly.chinacdc.cn/).

Based on published scientific evidence and expert opinions, potential environmental factors associated with HFRS transmission were collected for statistical analysis. These factors included meteorological, ecological, and socioeconomic variables. Firstly, meteorological factors (such as temperature, relative humidity, rainfall, wind speed, and sunshine index) were collected from the China Meteorological Data Sharing Center (http://data.cma.cn/) for the period between January 2004 and December 2019. Then, we collected monthly average concentration data on ecological variables such as forest cover, crop area, normalized difference vegetation index (NDVI), and the surface water resources from the China Environment Statistics Yearbook (https://www.mee.gov.cn/hjzl/). Finally, we computed the annual mean of meteorological and ecological variables using ArcGIS 10.8 software (ESRI Inc., Redlands, CA). For the annual socio-economic factors of 31 provincial-level administrative units from 2004 to 2019, gross domestic product (GDP), population density, urbanization rate, average years of education, and the number of medical personnel were collected from the China Statistical Yearbook (http://www.stats.gov.cn/).

### 2.2 Statistical analysis

#### 2.2.1 Age-period-cohort (APC) model

Age-Period-Cohort (APC) model was widely used to analyze infectious disease incidence and mortality trends. Based on the Poisson distribution, the model can estimate the risk of disease or death in a given population while adjusting for age, period, cohort, and other relevant factors [15]. Our study used the APC model to synchronously evaluate the age, period, and cohort effects of trends in 16 periods (2004–2019), and 16 age groups (0–4, 5–9, 10–14…,65–69, 70–74, 75 and above). Assuming that the count of HFRS incidences follows a Poisson distribution, the equation of the APC model is as follows:

(1) $$\log\left(R_{\mathrm{ijk}}\right)=\mu +\alpha \times \mathrm{Ag}e_{i}+\beta \times \mathrm{Perio}d_{j}+\gamma \times \mathrm{Cohor}t_{\kappa }+\varepsilon_{\mathrm{ij\kappa}}$$

where $R_{\mathrm{ijk}}$ represents the HFRS incidence of different groups, α, β and γ represent the regression coefficients of the age, period, and cohort effects of the model, respectively. μ represents the overall mean effect. $\mathrm{Ag}e_{i}$ represents the effect of the age group *i* (*i =* 1,2,3...16). $\mathrm{Perio}d_{j}$ is the effect of the period *j* (*j =* 1,2,3...16). $\mathrm{Cohor}t_{\kappa }$ represents the effect of cohort group $\kappa \;\left(\kappa =j-i+n\;\right)$ and n is the number of age groups. $\varepsilon_{\mathrm{ij\kappa}}$ denotes the residual of the APC model. The parameters (α, β, and γ) were exponentially transformed to represent the relative risk (*RR*) of a given age, period, and birth cohort relative to each average level. (*RR* > 1.0 indicates a higher risk relative to the average, and *RR* < 1.0 indicates a lower risk.) The analysis was performed using the “APCG1” package in the R software (version 4.1.3).

#### 2.2.2 Bayesian space-time hierarchy model (BSTHM)

In the study, we utilized BSTHM to identify the spatiotemporal characteristics of HFRS incidence in 31 provinces across mainland China from 2004 to 2019. The BSTHM for HFRS with Poisson distribution was used to evaluate the count of cases *y*_*it*_ and the potential risk population *n*_*i*_ as $y_{\mathrm{it}}∼\mathrm{Poisson}\left(n_{\mathrm{it}}u_{\mathrm{it}}\right)$. The underlying risk $u_{\mathrm{it}}$ is modelled as follows:

(2) $$\log\left(u_{\mathrm{it}}\right)=\alpha +s_{i}+\left(b_{0}t^{*}+v^{t}\right)+b_{1i}t^{*}+\varepsilon_{it}$$

where *u*_*it*_ represents the spatial relative risk (RR) of HFRS incidence for each province *i* (*i* = 1, …,31) and year *t* (*t* = 1, …,16), with *α* as the constant term, and the index *s*_*i*_ is the spatial disease risks in province *i*, which are influenced by certain relatively stable factors during the study period, including the local geographical environment, natural conditions, and economic level. The temporal term $\left(b_{0}t^{*}+v^{t}\right)$ describes the overall temporal trend and $t^{*}$represents the middle of the study period. The parameter *b*_*1i*_ captures the local trend in province *i,* and describes the spatial heterogeneity of a time trend. The term $\varepsilon_{\mathrm{it}}$ is the Gaussian random noise variable that indicating a random time effect.

Specific two-phase criteria were utilized to identify the spatial heterogeneity of relative risk for HFRS, and local temporal trends were explored based on the model's posterior estimation parameters. In the first phase, a province was defined as a hot spot for posterior probability *p* (exp (*s*_*i*_) > 1 | data) was more significant than 0.8 and as a cold spot if p (exp(*s*_*i*_) > 1 | data) was <0.2 [16]. The remaining provinces were categorized as warm spots. In the subsequent phase, the provinces corresponding to each risk group in the initial stage were further divided into three trend patterns based on the posterior estimated probability of the local province exp. (*b*_*1i*_). An increasing trend relative to the overall trend was assumed to be present if *p* (*b*_*1i*_ > 0|*hi*, data) ≥ 0.8, if it was <0.2, then a decreasing trend compared to the overall trend present, a local trend not differing from the overall trend if 0.2 < *p* (*b*_*1i*_ > 0|*hi*, data) < 0.8. Thus, this two-stage BSTHM formed a total of nine categories (three risk categories multiplied by three trend categories). All analyses were conducted utilizing Win BUGS software (Version 1.4.3, University of Cambridge, United Kingdom).

#### 2.2.3 Optimal parameters-based geographical detector (OPGD) model

The OPGD model has been applied to analyze the spatial variance, which can determine the most effective combination of parameters for assessing the predictive power of explanatory variables. Utilizing Q-statistics, this approach offers spatiotemporal perspectives and stratified heterogeneity of HFRS influence [17]. The Q value for an influential variable v is expressed as follows:

(3) $$Q=1-\frac{\sum_{j=1}^{M}N_{v,j}\sigma_{v,j}^{2}}{N_{v}\sigma_{v}^{2}}$$

where Qrepresents the determining power of influencing factors, its value ranges from 0 to 1, denoting the determinant power of a risk variable or a target factor's heterogeneity. *j* is the number of strata for different explanatory variables and *M* is the optimal strata parameter of explanatory variables based on the discretization methods in the OPGD model. *N*_*v*_ and $\sigma_{v}^{2}$ represent the total number of units and variance for HFRS in the whole study area, *N*_*v,j*_ and $\sigma_{v,j}^{2}$ are the numbers of units and variance of HFRS within the *j* th (*j = 1,..M*) sub-regions of variable *v.* The “GD” package within R software (Version 4.1.3) was used for all analyses.

## 3 Results

### 3.1 Epidemiological features and demographic characteristics

**Fig. 1:**
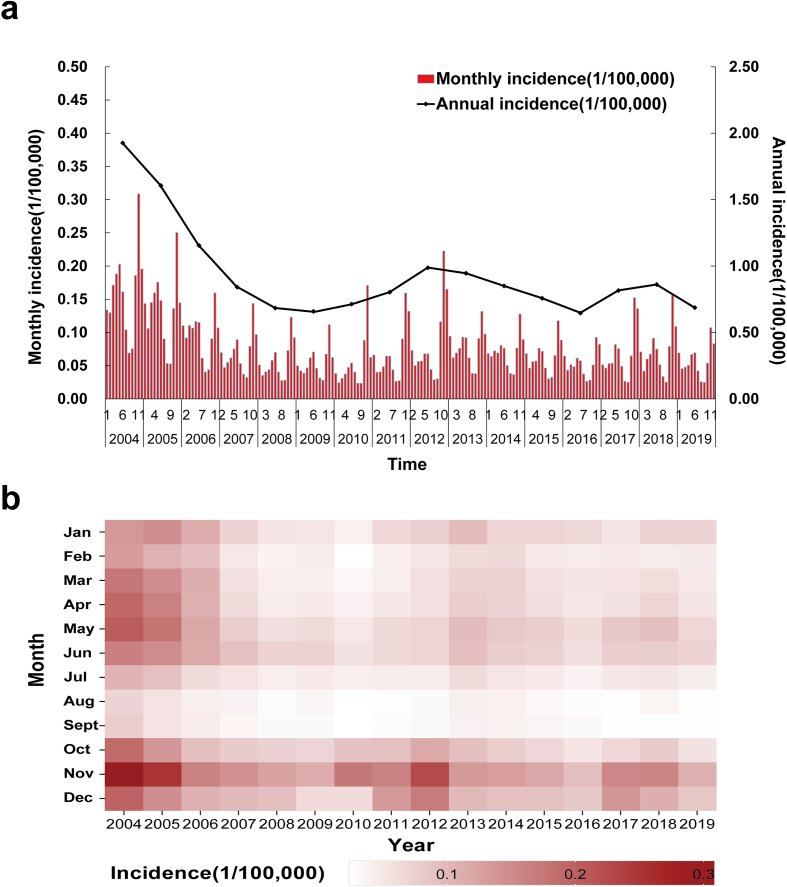
Temporal distribution of hemorrhagic fever with renal syndrome in China, 2004–2019. (a) The incidence of hemorrhagic fever with renal syndrome between 2004 and 2019. (b)The seasonal distributions of monthly incidence of hemorrhagic fever with renal syndrome.

From 2004 through 2019, mainland China documented 199,799 cases of HFRS with an annual incidence of 0.93/100,000 and annual mortality of 0.01/100,000. The annual incidence of HFRS showed a decreasing trend, gradually decreasing from 2004(1.93/100,000) to 2019(0.69/100,000) (Fig. 1a). The seasonal distribution of HFRS presented a bimodal pattern, mainly clustered in spring (March to June) and autumn-winter (October to January). The highest peak of incidence was reported in the autumn-winter period (October to January) (Fig. 1b).

**Fig. 2:**
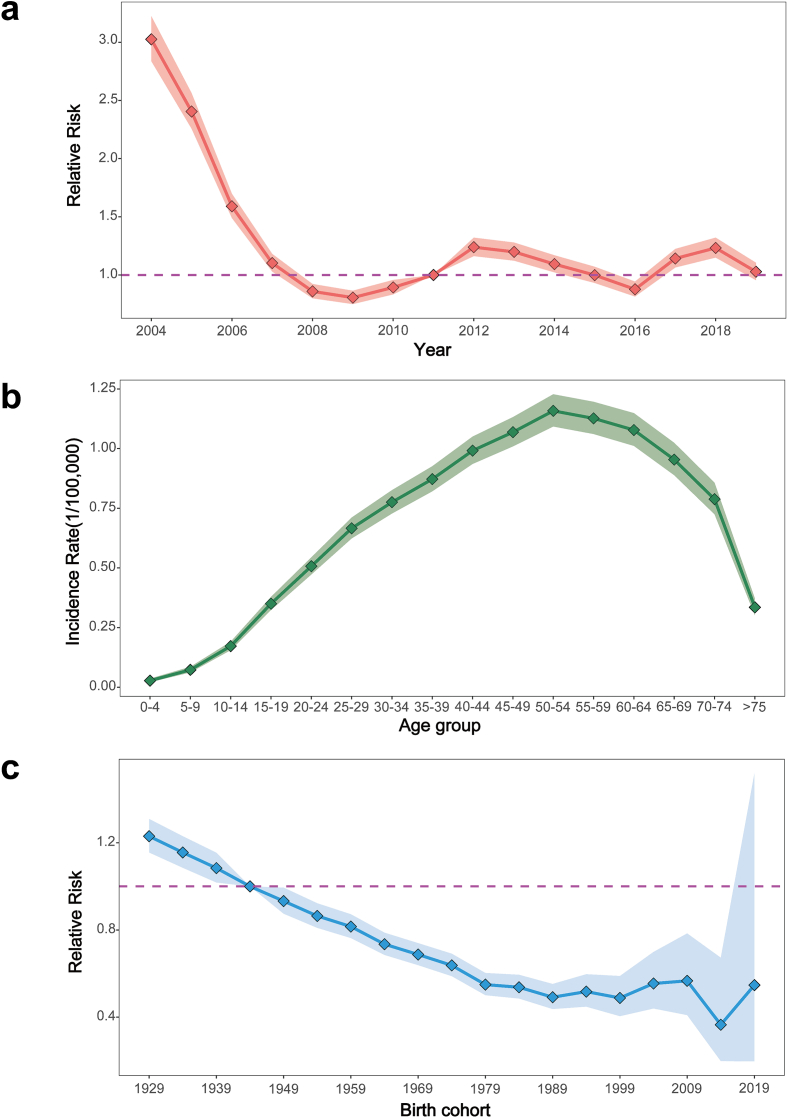
Temporal relative risk and risk groups of hemorrhagic fever with renal syndrome in China, 2004–2019. (a) The yearly temporal relative risk of hemorrhagic fever with renal syndrome in China, 2004–2019. (b)The variations of age groups in the incidence of hemorrhagic fever with renal syndrome. (c) The effects of the birth cohort on the relative risk of hemorrhagic fever with renal syndrome.

The results of the APC model indicated a significant decline trend in the temporal relative risk of HFRS in China from 2004 (RR = 3.02, 95% confidence interval [CI] = 2.84–3.22) to 2007 (RR = 1.10, 95% CI = 1.03–1.18). From 2008 to 2019, the RR value of HFRS fluctuated around 1.00. The incidence of HFRS exhibited a fluctuating, downward trajectory in China (Fig. 2a). Fig. 2b and Fig. 2c demonstrated the variation in HFRS incidence in China over the study period across different ages and birth cohorts. The incidence demonstrated an inverted U-shaped trend with advancing age, peaking in the 50–54 age group, with higher incidence observed among individuals aged 20–74 years. The 50–54 age group with the highest risk of incidence is approximately 42 times higher than the 0–4 age group with the lowest risk of incidence (Fig. 2b). Birth cohort effects showed that the risk of HFRS decreased with a later date of birth (Fig. 2c). The birth cohort (1929–1933) had the highest risk of HFRS (RR = 1.23, 95% CI = 1.16–1.31), and these cohorts after 1944 were significantly lower than the overall average risk of HFRS.

### 3.2 Spatial-temporal heterogeneity

**Fig. 3:**
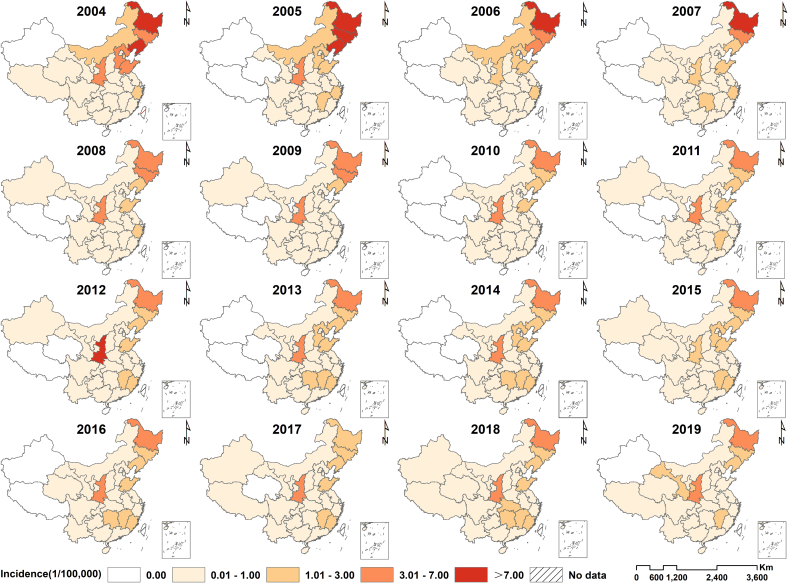
Spatial and temporal distributions of hemorrhagic fever with renal syndrome at the provincial level in China, 2004–2019.

**Fig. 4:**
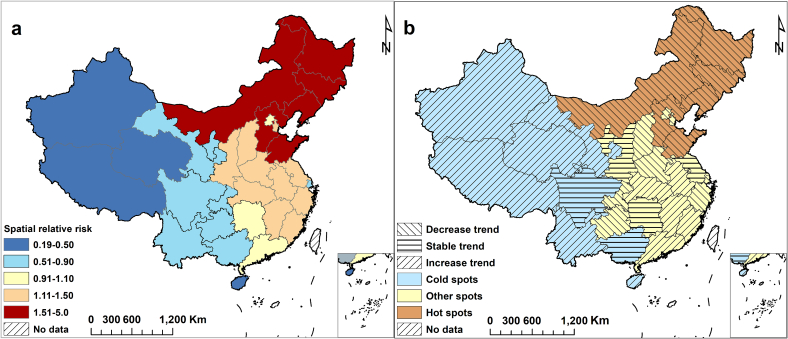
Spatial-temporal heterogeneity of hemorrhagic fever with renal syndrome in mainland China, 2004–2019. (a) The spatial relative risks of hemorrhagic fever with renal syndrome at the provincial administrative units, (b) The distribution of hot and cold spots, and province-level temporal trends of hemorrhagic fever with renal syndrome.

Fig. 3 illustrates the spatial-temporal distribution of the annual incidence of HFRS in China from 2004 to 2019 at provincial levels. There were clear geographical differences in the yearly incidence of HFRS, with rates ranging from 0 to 13.05 per 100,000. Since 2004, the number of provinces with a high incidence(> 1.00 per 100,000) has been shrinking. During the study period, the provinces with a high incidence of HFRS were Hebei, Liaoning, Jilin, Heilongjiang, Jiangxi, Shandong, and Shaanxi. Based on the results calculated using BSTHM, Fig. 4a displays the relative spatial risks of HFRS at the provincial level from 2004 to 2019. The Northeastern provinces of China exhibited higher relative spatial risks, thereby indicating an increased risk of HFRS in these regions. The relative spatial risks of HFRS were comparatively low across all provinces in the central and western regions.

Based on the results of the first-stage criterion that utilized the posterior estimated parameters of BSTHM, the 31 provinces in China were categorized into three groups: hot spots, warm spots, and cold spots. Specifically, 6 (19.35%) provinces were identified as hot spots, 8 (25.81%) provinces as cold spots, and the other 17 provinces (54.84%) as warm spots. As shown in the Fig. 4b, the hot-spot regions were mainly distributed in northeast and north China. In contrast, the cold-spot regions were primarily distributed in western China.

The analysis of time trends, using the second stage standard, indicates that six hot-spot provinces, including Liaoning, Jilin, Heilongjiang, and Inner Mongolia, potentially exhibit a downward trend as compared to the overall trend. As a result, the risk in these provinces may presently be higher than the overall risk, but it is expected to be lower in the future. Consequently, these provinces may become lower risk regions (Fig. 4b). Among the eight cold-spot provinces such as Hainan, Yunnan, Tibet, Gansu, Qinghai, and Xinjiang exhibit an increasing trend, whereas Guangxi and Sichuan displayed a stable trend and will remain low-risk areas in the future. In addition, among the 17 provinces are warm-spot regions, such as Anhui, Hubei, Jiangxi, Fujian and Guangdong are experiencing an upward trend and may transition into higher risk provinces or turn into hot-spot provinces over time. Meanwhile, Beijing, Tianjin, Shanxi, Henan, Shanghai, Zhejiang, Chongqing and Guizhou are all displaying a decreasing trend, suggesting that these 8 provinces are likely to become cold-spot regions over time.

### 3.3 Risk factors of HFRS

In the OPGD model, a total of 18 potential factors were collected as the explanatory variables of HFRS incidence in China, including eight socio-economic factors (per capita GDP, population density, urbanization rate, education level, medical level, city drainage pipe length, the index of night light, and built-up area), five ecological factors (NDVI, forest cover, crop area, the surface water resources and area of forest rodent infestation), and five meteorological factors (temperature, rainfall, wind speed, relative humidity, and sunshine index) (Table 1). All variables were used to assess the determinant power of these factors on HFRS incidence.

**Table 1:** Summary of meteorological factors, ecological factors and socio-economic factors in China, 2004–2019.

Variable		Average	5% percentile	50% percentile	95% percentile
Meteorological factors	Temperature (°C)	13.66	4.10	14.83	22.33
	Rainfall (mm)	8402.66	3940.63	8365.23	17,760.15
	Wind speed (m/s)	2.15	1.56	2.17	2.83
	Relative humidity (%)	66.55	50.49	67.64	80.07
	Sunshine index (h)	2070.20	1206.79	2073.62	2837.85
Ecological factors	NDVI	0.67	0.32	0.75	0.82
	Forest cover (%)	32.40	5.60	34.90	60.20
	Crop area (thousand hectares)	5179.11	277.06	4600.41	13,755.59
	Area of forest rodent infestation (hectares)	370,638	19,805	319,700	1,052,750
	The surface water resources (100 million cubic meters)	850.68	8.47	519.40	2466.47
Socio-economic factors	Per capita GDP (10,000 RMB)	4.03	0.91	3.54	9.70
	Population density (persons/sq.km)	431.36	7.92	272.16	1292.44
	Urbanization rate (%)	52.31	29.08	51.29	96.01
	Education level (Educated year)	8.64	6.80	8.69	10.69
	Medical level (10,000 medical workers)	21.67	2.39	18.99	53.01
	City drainage pipe length (km)	1.42	0.11	0.99	4.71
	The index of night light	5.91	0.12	2.87	23.46
	Built-up area (km^2^)	1446.97	162.95	1201.50	4091.35

**Fig. 5:**
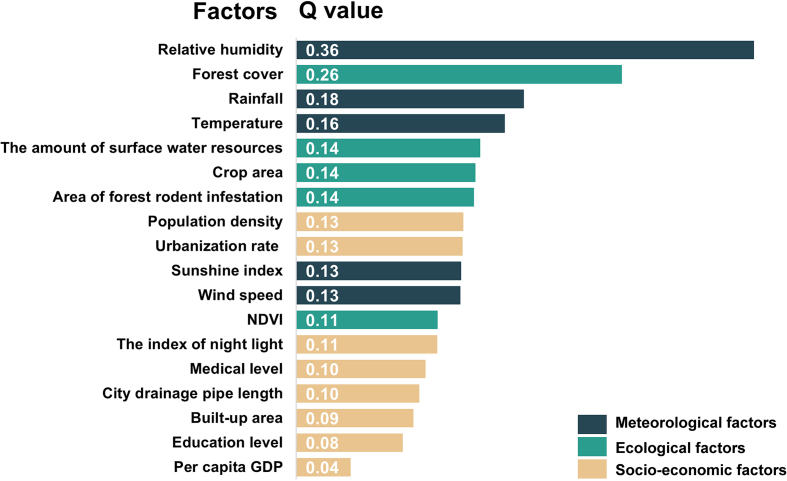
The determinant power of each factor in the spatial-temporal distribution of hemorrhagic fever with renal syndrome in China, 2004–2019.

As depicted in Fig. 5, the meteorological and ecological factors exerted the most significant impact on the spatial-temporal variations of HFRS in China. The relative humidity showed the strongest association with the incidence of HFRS (Q value = 0.36, *P* < 0.01). Followed by the forest cover (Q value = 0.26, P < 0.01), rainfall (Q value = 0.18, P < 0.01), temperature (Q value = 0.16, P < 0.01), the surface water resources (Q value = 0.14, P < 0.01), crop area (Q value = 0.14, P < 0.01) and area of forest rodent infestation (Q value = 0.14, P < 0.01). In contrast, the city drainage pipe length, built-up area, education level, and per capita GDP had relatively lower effects on the distribution of HFRS, with Q values of 0.10, 0.09, 0.08, and 0.04, respectively.

**Fig. 6:**
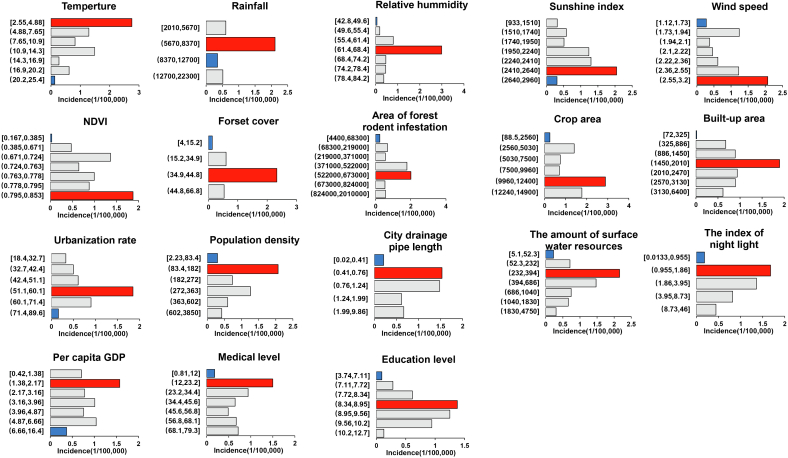
The different determinant effect of each factor in the spatial-temporal distribution of hemorrhagic fever with renal syndrome in China, 2004–2019.

We found that each influencing factor's different levels had varying determinant effects on HFRS incidence at the provincial level of China. In Fig. 6, red indicates that this influencing factor contributes the most to the incidence of HFRS in this interval, and blue indicates that this influencing factor contributes the least to the incidence of HFRS in this interval. The ordinate interval of the histogram is divided into approximate equals according to the maximum and minimum contribution intervals. Low levels of socioeconomic factors, such as the index of night light, per capita GDP, and the medical level were closely associated with a higher incidence of HFRS. Meanwhile, high wind speed, NDVI, forest cover and crop area were tightly related to the high incidence of HFRS.

Based on the OPGD model, we found significant heterogeneity in the main determinants across regions. In the hot-spot regions, the top five determinants were per capita GDP, the index of night light, forest cover, population density, and NDVI with a Q value of 0.49, 0.48, 0.46, 0.44, and 0.37, respectively. While the HFRS was significantly influenced by several factors in the warm-spot regions, including relative humidity (Q = 0.33), population density (Q = 0.28), temperature (Q = 0.28), wind speed (Q = 0.26), and crop area (Q = 0.25) (Table 2).

**Table 2:** The top 5 determinant factors and specific Q value in different regions in China, 2004–2019.

Rank	Hot-spot regions		Warm-spot regions	
	Variables	Q value	Variables	Q value
1	Per capita GDP	0.49	Relative humidity	0.33
2	The index of night light	0.48	Population density	0.28
3	Forest cover	0.46	Temperature	0.28
4	Population density	0.43	Wind speed	0.26
5	NDVI	0.37	Crop area	0.25

## 4 Discussion

China, recognized as the typical area with the highest incidence of HFRS, has reported 570,000 cases from 1995 to 2020 alone, posing a significant threat to public health. Despite a general declining trend in recent years due to effective large-scale vaccination and other prevention measures, localized outbreaks persist, maintaining high incidence levels. Therefore, investigating the long-term prevalence trends and spatio-temporal characteristics of HFRS, and identifying key factors driving variations in its temporal and spatial attributes can offer essential scientific guidance for targeted epidemic prevention and control strategies in high-risk regions. Our findings were as follows: (1) The epidemic of HFRS in China has exhibited a declining trend and appears to be approaching stability. (2) The majority of patients were middle-aged and elderly individuals, with the peak incidence observed in the 50–54 age group. (3) Notably, distinct variations in incidence were observed across different geographic areas, with northeast China experiencing high incidence clusters, all of which displayed a decreasing trend. (4) Overall, the impact of meteorological factors on the occurrence of HFRS is notably stronger than that of ecological and socio-economic factors.

The annual incidence of HFRS in China decreased from 2004 to 2009, and then fluctuated, which is consistent with the previous study [18]. During our temporal analysis using the APC model, the research findings revealed a significant decline trend in the risk of HFRS, especially from 2004 to 2009. The continued decline that followed could most likely be credited to a set of control measures for HFRS in China, alongside advancements in social, environmental, and living conditions. Based on the monthly analysis, it was discovered that the incidence of HFRS displayed a bimodal distribution throughout the study period. The average monthly incidence was >0.12/100,000 in the fall and winter months (October–December) and >0.08/100,000 in the spring months (March–June). Similar temporal patterns have been observed in various regions of China by other studies [19]. This implies that control measures should be implemented during the fall, winter, and spring seasons to avoid increased morbidity. It is also worth noting that there are still about 10,000 cases per year since 2008, which emphasizes the need for continued attention to HFRS [20].

The results of the APC model indicate that there was an unimodal age effect observed across 16 age groups. Similar to previous findings, it was discovered that the highest occurrence of HFRS was in the age group of 50–54 [21]. Also, the relative risk of age groups 40–64 years old (birth cohort groups after 1954 and before 1979) was much higher than that of age groups <15 years old (birth cohort groups after 2004), which was consistent with the previous studies [22]. The elevated risk for the elderly population may be primarily attributed to rat exposure rates and compromised immune system function. The incidence of HFRS is influenced by the rate of rat exposure, with individuals aged 40–64 experiencing the highest incidence. This phenomenon may be attributed to the significant migration of rural young and middle-aged individuals to urban areas for employment, leaving behind an elderly population that has become the primary workforce in agricultural production activities. Consequently, this has led to an increased vulnerability of the elderly to exposure risks [23]. Therefore, the primary focus is on identifying, safeguarding, and treating cases within high-risk age groups in HFRS endemic areas, and adjusting the HFRS vaccination program to include individuals aged 61–74 promptly based on the prevailing circumstances [24].

The study revealed significant spatial heterogeneity in the risk of HFRS across China. Provinces with the high risk of HFRS were primarily distributed in north China (Hebei), northeast China (Liaoning, Jilin, Heilongjiang), eastern China (Jiangxi, Shandong), and northwestern China (Shaanxi). This is consistent with previous research, the majority of regions with high HFRS incidence are situated in the eastern, northeastern, and central parts of China, mainly in temperate and subtropical climate zones [25]. Evidence suggests that the distribution of HFRS epidemic areas in China is characterized by natural lesions occurring mainly in plain and hilly areas at elevations below 500 m above sea level in the eastern monsoon region of China, as well as in temperate and subtropical areas with abundant water [26]. Although the annual incidence of HFRS decreased significantly from 2004 to 2019 throughout China, extensive regional variations were detected in trend analysis among the 31 provinces. Most provinces experienced a declining trend and have potentially become regions with lower risk in comparison to the overall trend. The decline could be credited to effective public health interventions and intensified control strategies for HFRS in recent years [27]. However, several provinces showed an increasing trend through our analysis, especially the five warm-spot regions (Anhui, Fujian, Jiangxi, Hubei, and Guangdong). These five regions still had higher risks of HFRS during the study period, and would likely remain hot-spot regions in the future. These provinces have significant populations and are experiencing swift urbanization. Epidemiological evidence suggests that the regions with the highest likelihood of human infection are those with a high incidence of migrant populations and dense working and living conditions, notably inadequate housing, leading to frequent close contact with infected rodents' excreta [28]. Additionally, seven provinces in cold-spot areas (Guangxi, Hainan, Yunnan, Tibet, Gansu, Qinghai, and Xinjiang) showed a potential possibility of becoming warm-spot regions. This increase may be due to increased rainfall in northwestern China in recent years, resulting in increased relative humidity and forest cover. At the same time, HFRS is a climate-sensitive infectious disease controlled by a variety of socio-economic factors, and rainfall and humidity have been confirmed as driving factors of HFRS [29]. We suggested that these areas should be aware of the increased risk of the HFRS.

As a typical zoonotic disease, the transmission and epidemic of HFRS have specific demographic, geographical and seasonal distribution characteristics, and these characteristics are mainly affected by host animals, meteorological factors, ecological environment, and human production activities. Similar to previous studies our analysis results also presented that the meteorological factors were the dominant influencing factors of the spatial-temporal variations in the prevalence of HFRS, while ecological and socio-economic factors had relatively slighter contributions to the incidence of HFRS [30]. Meteorological factors significantly impact the spread and prevalence of HFRS by affecting the food supply and living environment of host animals. This leads to changes in population density, living habits, and HV infection activity of host animals, influencing the scale of HFRS outbreaks [31]. As with previous studies suggest that HFRS risk may be strongly associated with low temperatures, moderate rainfall, moderate relative humidity and high wind speeds [32]. However, it should be noted that there are varying regional differences in the transmission of the HFRS epidemic, even when affected by the same factor. For example, the occurrence of HFRS in the northeast displayed a positive correlation with precipitation, whereas in Qingdao, there was a negative correlation between the two factors [33]. Ecological factors similarly influence the transmission and prevalence of HFRS primarily by altering the distribution and density of host animal populations. French researchers discovered a strong correlation between the Normalized Difference Vegetation Index (NDVI) and the intensity of the HFRS epidemic in eastern France's Franche-Comté region during the period of 1999 to 2008. This was in line with our study's findings that regions with high forest cover and NDVI levels exhibit an increased risk of HFRS [34]. Given the crucial role of animal host transmission in HFRS outbreaks and the significance of predicting meteorological and ecological factors, early surveillance focusing on rats as the primary vector is recommended. This approach includes implementing comprehensive prevention measures to deter and eliminate rats, enhancing rodent epidemic surveillance, fostering inter-departmental collaboration for prevention and control, intensifying efforts to reduce rodent populations, and addressing the root cause to prevent and manage HFRS outbreaks effectively.

Moreover, the findings of our study suggest that socio-economic factors may exhibit a comparatively weaker correlation when compared to other determinant categories. Indicators such as the index of night light, per capita GDP, urbanization rate, educational level, and medical level are commonly employed to gauge the level of social development within a province. Regions with high population density and urbanization rates may have an elevated risk of HFRS, in accordance with our research. Previous studies have provided a clear interaction between HFRS incidence and socio-economic factors, especially a positive correlation between urbanization rates and GDP [35]. In addition, rapid urbanization will accelerate the expansion of farm and pasture areas. The expansion of farmland and pasture can create preferred habitats for rodents, resulting in increased rat density and an increased risk of infection. Previous studies have also demonstrated a strong correlation between the uneven regional economic development and the spatial distribution of HFRS risk in China. The study revealed that there is heterogeneity in the determinant factors of HFRS risk across different regions. In hot-spot regions, the three main contributing factors to power are per capita GDP, the index of night light, and forest cover. While the relative humidity, the population density, and the temperature showed the most importance on HFRS incidence in the warm-spot regions. This variability likely resulted from varying degrees of economic growth, living conditions, geographic features, and intensity of prevention and control measures against HFRS across different regions. This variability of factors in different regions requires further research in the future.

In conclusion, this study systematically examined the epidemic characteristics, temporal and spatial trends of HFRS in China spanning from 2004 to 2019. The research identified the driving factors contributing to regional disparities in HFRS incidence. By evaluating the current prevalence of HFRS and its distribution across endemic regions, this research offers a scientific foundation for health authorities to implement targeted and efficient prevention and control strategies. Furthermore, it underscores the importance of enhancing surveillance and reporting mechanisms, increasing public awareness, and administering vaccinations to individuals aged 15–74 in high-incidence areas and high-risk groups with frequent exposure to rodents in their occupational and daily environments.

Some limitations of the study need to be acknowledged. First, owing to the unavailability of data, we were unable to collect detailed demographic information including sex, occupation, and residence that would have enabled us to explore the detailed epidemic features. In addition, our ecological model evaluated the association between potential factors and the spatiotemporal occurrence of HFRS, while the overall transmission pathway of hantavirus is expected to be more complex. Finally, our study did not include other factors such as vaccination data, public health initiatives, and personal protective measures. These limitations may be addressed in future studies that are better designed.

## 5 Conclusions

This study provides a detailed overview of the incidence of HFRS in various Chinese provinces from 2004 to 2019. It distinguishes regions with increased risk and factors influencing the disease transmission, and may provide valuable insights for the development of prevention and control strategies.

## Ethical approval

The data we analyzed was publicly available materials which not included private information of cases, so the work could not need a ethical approval.

## Consent to participate

All authors contributed to the study conception and design.

## Consent to publish

All the authors gave their consent for this article to be published.

## Funding

This work was supported by the 10.13039/501100001809National Natural Science Foundation of China (grant number: 82273689 and 81803289).

## CRediT authorship contribution statement

**Bo Wen:** Data curation, Formal analysis, Writing – original draft, Writing – review & editing. **Zurong Yang:** Data curation, Formal analysis, Writing – review & editing. **Shaolong Ren:** Writing – review & editing. **Ting Fu:** Writing – review & editing. **Rui Li:** Data curation. **Mengwei Lu:** Methodology. **Xiaoang Qin:** Formal analysis. **Ang Li:** Formal analysis. **Zhifu Kou:** Formal analysis. **Zhongjun Shao:** Funding acquisition, Resources, Writing – review & editing. **Kun Liu:** Conceptualization, Data curation, Writing – review & editing.

## Declaration of competing interest

The authors declare that the research was conducted in the absence of any commercial or financial relationships that could be construed as a potential conflict of interest.

## Data Availability

Data and materials will be made available on request to the authors.
